# Intrafraction motion and impact of margin reduction for MR‐Linac online adaptive radiotherapy for pancreatic cancer treatments

**DOI:** 10.1002/jmrs.832

**Published:** 2024-10-13

**Authors:** Ashleigh Fasala, Madeline Carr, Yolanda Surjan, Parmoun Daghigh, Jeremy de Leon, Abbey Burns, Vikneswary Batumalai

**Affiliations:** ^1^ GenesisCare Sydney New South Wales Australia; ^2^ College of Health, Medicine and Wellbeing, School of Health Sciences, Global Centre for Research and Training in Radiation Oncology The University of Newcastle Callaghan New South Wales Australia; ^3^ School of Physics University of Sydney Sydney New South Wales Australia; ^4^ The George Institute for Global Health UNSW Sydney Sydney New South Wales Australia

**Keywords:** Abdominal compression, intrafraction motion, margin reduction, MR‐Linac, pancreatic cancer, stereotactic ablative body radiotherapy

## Abstract

**Introduction:**

Online adaptive radiotherapy is well suited for stereotactic ablative radiotherapy (SABR) in pancreatic cancer due to considerable intrafractional tumour motion. This study aimed to assess intrafraction motion and generate adjusted planning target volume (PTV) margins required for online adaptive radiotherapy in pancreatic cancer treatment using abdominal compression on the magnetic resonance linear accelerator (MR‐Linac).

**Methods:**

Motion monitoring images obtained from 67 fractions for 15 previously treated pancreatic cancer patients were analysed. All patients received SABR (50 Gy in five fractions) on the MR‐Linac using abdominal compression. The analysis included quantification of intrafraction motion, leading to the development of adjusted PTV margins. The dosimetric impact of implementing the adjusted PTV was then evaluated in a cohort of 20 patients.

**Results:**

Intrafraction motion indicated an average target displacement of 1–3 mm, resulting in an adjusted PTV margin of 2 mm in the right–left and superior–inferior directions, and 3 mm in the anterior–posterior direction. Plans incorporating these adjusted margins consistently demonstrated improved dose to target volumes, with improvements averaging 1.5 Gy in CTV D99%, 4.9 Gy in PTV D99% and 1.2 Gy in PTV‐high D90%, and better sparing of the organs at risk (OAR).

**Conclusions:**

The improved target volume coverage and reduced OAR dose suggest potential for reducing current clinical margins for MR‐Linac treatment. However, it is important to note that decreasing margins may reduce safeguards against geographical misses. Nonetheless, the continued integration of gating systems on MR‐Linacs could provide confidence in adopting reduced margins.

## Graphical abstract

This analysis included quantification of intrafraction motion in pancreas stereotactic ablative radiotherapy treated on the MR‐Linac, leading to the development of adjusted (PTV) margins. Intrafraction motion indicated an average target displacement of 1–3 mm, resulting in an adjusted PTV margin of 2 mm in the right–left and superior–inferior directions, and 3 mm in the anterior–posterior direction.

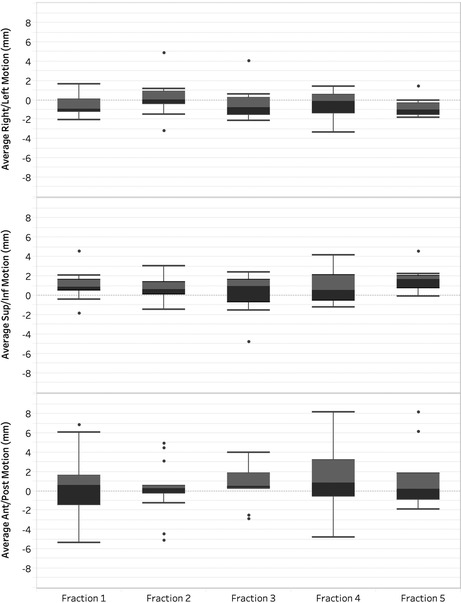

## Introduction

Pancreatic cancer is the 12th most prevalent cancer globally,^1^ with approximately 49% of cases warranting consideration for radiotherapy.^2^ Despite its prevalence, the survival rate for pancreatic cancer has remained low with a 5‐year overall survival (OS) of only 12%.^2^ Part of the reason for this poor prognosis is that pancreatic cancer is often diagnosed at a late stage, as its symptoms, such as abdominal pain, weight loss and jaundice, are frequently misattributed to more common, less severe conditions, delaying diagnosis and treatment. The disease significantly impacts patients' quality of life (QOL), often due to its aggressive nature and side effects that result from the close proximity of the pancreas to critical surrounding organs.^3^ Treatments, including both radiotherapy and chemotherapy, can lead to severe toxicities, further lowering QOL.^4^, ^5^ These toxicities can include gastritis, duodenal bleeding, nausea, vomiting and, in the worst cases, bowel obstructions.^6^ However, novel technology and imaging methods have created a unique opportunity for radiotherapy to contribute to improvements of survival outcomes.

Stereotactic ablative body radiotherapy (SABR) has emerged as a focal point in the exploration of treatment strategies for locally advanced pancreatic cancer (LAPC). Investigations suggest that escalation of the biologically effective dose (BED) ≥ 100 Gy may improve local control (LC) and OS for patients with LAPC.^7^, ^8^, ^9^, ^10^, ^11^, ^12^, ^13^ However, achieving this dose in pancreatic cancer treatment poses challenges due to the organ's proximity to gastrointestinal organs and the impact of intrafraction motion. Although cone beam computed tomography (CBCT) and tracking of fiducial markers using conventional linear accelerator may improve treatment precision, the anatomical location of the pancreas results in significant volume deformation, limiting treatment accuracy.^14^ The use of CBCT often poses limitations in soft‐tissue visualisation, complicating the accurate assessment of tumours and surrounding organs at risk (OAR). The advent of the magnetic resonance linear accelerator (MR‐Linac) can help overcome these challenges, offering high‐contrast imaging of tumours and OARs, thereby better enabling precise implementation of dose escalation and adaptive strategies. Recent studies have demonstrated higher survival rates and lower incidences of severe toxicity with MR‐Linac‐guided SABR for LAPC.^12^, ^15^, ^16^, ^17^

The substantial treatment margin required as a result of intrafraction motion^18^ may result in high dose to the surrounding OARs.^14^ More recently, the management of intrafraction motion through abdominal compression for LAPC has been shown to be a promising approach, significantly reducing tumour motion by restricting respiratory movement.^19^, ^20^, ^21^ Leveraging the capabilities of MR‐Linac technology along with abdominal compression holds the potential for precise margin reduction to effectively target and treat LAPC while sparing the healthy tissue from damaging high doses.

This study aimed to assess intrafraction motion and generated adjusted PTV margins required for online adaptive radiotherapy in pancreatic cancer treatment using abdominal compression on the MR‐Linac. Additionally, this study aimed to explore the dosimetric implications of reducing PTV margins in the delivery of pancreatic cancer treatment.

## Methods

### Patients

The study cohort comprised patients previously treated for non‐operable LAPC on the Unity MR‐Linac (Elekta AB, Stockholm, Sweden) from December 2021 to July 2023. All patients were part of the GenesisCare Oncology Outcomes Project, approved by the St Vincent's Hospital Human Research Ethics Committee (reference number 2022/ETH00247). Two distinct patient cohorts were involved: the motion cohort, involving 15 LAPC patients for retrospective intrafraction motion assessment and adjusted PTV determination; and the dosimetry cohort, consisting of 20 patients to evaluate the dosimetric impact of the adjusted PTV margin. Images were excluded based on the following exclusion criteria: datasets with less than 2 min of cine motion images and fewer than four treatment fractions.

### Simulation, delineation and planning

Prior to treatment delivery, patients underwent MR simulation. A 3D‐Vane MR scan was first acquired using the MR‐Linac which served as the reference MR image. The 3D‐Vane sequence is a free‐breathing imaging strategy that suppresses motion artefacts by continuously acquiring data over the complete respiratory cycle, resulting in averaged imaging of moving targets and OARs.^22^ Patients were positioned supine in a vac‐bag and a ZiFix Abdominal/Thoracic Motion Control System compression belt, customised for each patient. Additionally, a computed tomography (CT) simulation scan (Siemens Somatom Definition AS; Siemens Healthineers, Erlangen, Germany) was acquired in the same position.

The gross tumour volume (GTV) contour was determined based on patients' treatment intent. The GTV was the visible primary tumour and included high‐risk areas, superior mesenteric artery and vein, coeliac axis and adjacent retroperitoneum. A separate GTV was outlined for any gross nodal volume. The clinical target volume (CTV) comprised both primary and nodal GTVs with a 0 mm margin. A 3–5 mm margin was applied around the CTV in each plane at the discretion of the radiation oncologist (RO) to create the planning target volume (PTV). OAR delineation included the duodenum, stomach, jejunum and ileum, colon and kidneys. A 3–5 mm expansion of the duodenum, jejunum, ileum, colon and stomach formed planning risk volumes (PRV), which were critical structures to be avoided during planning. Due to the proximity of PRVs to the PTV, a PTV‐high structure was created by subtracting any overlap between critical structures and the PTV.

Patients were prescribed to receive 50 Gy in five fractions (three fractions per week, one daily). To adhere to OAR constraints, any overlapping portion of the PTV by the PRVs was constrained to 33 Gy, while the remainder (PTV‐high) was escalated to 50 Gy. All plans were generated using Monaco (version 5.40.01; Elekta AB, Stockholm, Sweden) with a step‐and‐shoot intensity‐modulated RT (IMRT) plan with a maximum of 100 segments per plan and 2‐mm dose calculation grid. A standard plan template with 15 non‐uniformly spaced beam angles was used, and any beams deemed unnecessary were removed at the discretion of the radiation therapists and physicists. The dosimetric criteria for plan evaluation are outlined in Table 1.

**Table 1:** Plan compliance criteria.

Structure	Objective	Protocol	Minor violation	Major violation
Clinical target volume	D99%	>33 Gy	30–33 Gy	<30 Gy
Planning target volume (PTV)	D99%	>30 Gy	25 Gy	–
	Dmax	55–65 Gy	<55 Gy	>65 Gy
PTV‐high	D90%	>100%	90–99%	<90%
Duodenum	Dmax	<33 Gy	<35 Gy	>35 Gy
	V30 Gy	<5 cm^3^	5–10 cm^3^	>10 cm^3^
	V20 Gy	<20 cm^3^	20–40 cm^3^	>40 cm^3^
Stomach	Dmax	<33 Gy	< 35 Gy	>35 Gy
	V30 Gy	<5 cm^3^	5–0 cm^3^	>10 cm^3^
	V20 Gy	<20 cm^3^	20–40 cm^3^	>40 cm^3^
Jejunum and ileum	Dmax	<33 Gy	<35 Gy	>35 Gy
	V30 Gy	<5 cm^3^	5–10 cm^3^	>10 cm^3^
	V20 Gy	<20 cm^3^	20–40 cm^3^	>40 cm^3^
Colon	Dmax	<35 Gy	<35–38 Gy	>38 Gy
Kidneys	V12 Gy	<25%	25–30%	>30%
Left kidney	V10 Gy	<10%	10–25%	>25%
Right kidney	V10 Gy	<10%	10–25%	>25%
Liver	V12 Gy	<40%	<50%	>50%

Dmax, maximum dose; Dx%, dose received by x% of the structure; Gy, Grey; PTV‐high, PTV minus critical structures; VxGy, the percentage of the volume receiving more than xGy.

### SABR treatment

Patients underwent treatment using a standard online adaptive SABR protocol on the MR‐Linac, incorporating an adapt‐to‐shape (ATS) strategy for each fraction. Before the delivery of each fraction, a 3D Vane MR scan was acquired and rigidly registered to the reference MR image acquired during the simulation. Following this, deformable image registration was used to project the original set of contours onto the daily pre‐treatment MR image, and the target and OARs were recontoured by the radiation oncologist. Plan reoptimisation was completed, and treatment was initiated. During treatment, real‐time balanced turbo field echo (btFFE) 2D cine MR imaging was acquired to monitor intrafraction motion. The duration of the scans was determined by the stability of the tumour motion, as assessed by the treating staff. After treatment, all imaging data were exported for comprehensive motion quantification.

### Motion analysis

Intrafraction motion analysis of CTVs was conducted using a motion management research program (MMRP) (Elekta AB). To do this, the cine scans collected during treatment were imported to MMRP following the patient's treatment and used to determine new PTV margins. An exclusion criterion for including patient cine data was that scan durations had to be >2 min to acquire sufficient images for margin data collection. Insufficient statistics in motion data could introduce inaccuracies when interpolating the data to generate a new margin, as a lack of sufficient scan duration might not provide a reliable trend line for patient motion during treatment. Consequently, only cine scans with durations exceeding 2 min were included to ensure the accuracy and reliability of the motion analysis and subsequent margin determination. Each image dataset per fraction included a 3D Vane MR scan and corresponding CTV delineations, and a btFFE 2D cine motion scan.

To determine the PTV margins, manual rigid registration of the 3D MR scan and 2D cine motion images was first completed using the MMRP Template Reviewer application (Elekta AB). The registered data were then processed in MMRP to quantify the average intrafraction motion for each patient and fraction. As an overview, MMRP calculates the tumour's displacement in the 2D cine motion images relative to the stationary tumour position (as identified by the CTV delineation) in the registered 3D MR scan, over the total duration of the cine scan. The average displacement is measured in the right–left (RL), superior–inferior (SI) and anterior–posterior (AP) directions to quantify the total intrafraction motion. A detailed description of the software's functionality and motion quantification methods can be found in the literature.^23^, ^24^ The average CTV intrafraction motion values in each direction were then used to create the adjusted PTV margins. New calculated margins were inclusive of observed respiratory motion that was reduced by abdominal compression belts. While margins account for motion during treatment, intrafraction motion could cause tumours to briefly move outside these margins.^25^ The MMRP was also used to assess the percentage of time the tumour remained within specified margins during treatment imaging.

### Dosimetric analysis

The margin recipe outlined by Janssen et al.^26^ accounts for tumour motion and drift, assuming that setup errors and delineation errors are statistically independent, an assumption appropriate in the context of online adaptive planning with motion management. Tumour drift is the gradual, continuous shift of a tumour's position during treatment sessions. Unlike rapid movements caused by respiration or cardiac motion (tumour motion), tumour drift is a slower, progressive displacement due to various physiological factors (e.g. peristalsis). This margin recipe was adapted in developing adjusted PTV structures, while maintaining the same OAR and PRV margins as the original clinical plans. Subsequently, new reference plans were generated by incorporating the adjusted PTV margins for each patient in the dosimetry cohort. All new plans were assessed and approved by an experienced radiation therapist specialised in MR‐Linac treatment and planning. The new plans with adjusted margins were dosimetrically compared to the clinical reference plan.

## Results

**Figure 1:**
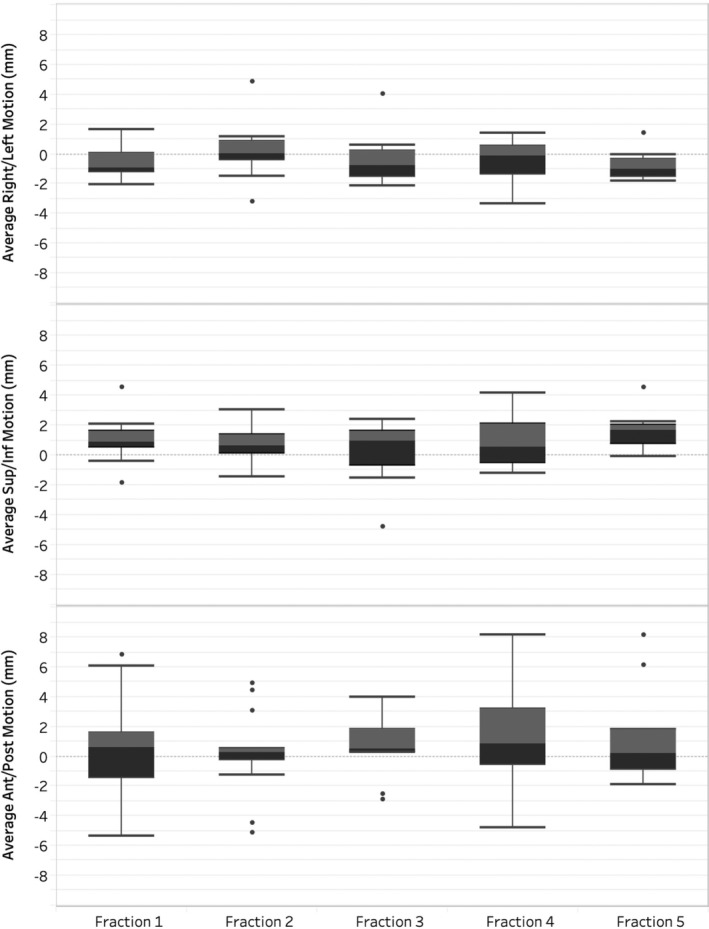
Box plots of intrafraction motion for all patients in right–left, superior–inferior and anterior–posterior planes. Each box plot represents the distribution of motion with the median depicted by the central line, the interquartile range (IQR) by the box and the whiskers showing 1.5 times the IQR. Outliers are indicated by individual points.

A total of 67 out of 75 datasets were analysed using MMRP. Eight datasets were excluded for not meeting the inclusion criteria: six had < 2 min of cine motion images, and two failed to process correctly through the MMRP. Figure 1 presents box plots of intrafraction motion across five fractions. The median motion is consistently around ±1 mm, with interquartile range within −2 mm to 2 mm. The AP dimension shows the greatest variability, with outliers reaching up to 8 mm.

Overall, the results indicate an average tumour motion of 1 mm in left and inferior directions, 2 mm in the right, superior and anterior directions and 3 mm in the posterior direction. Predominantly, motion in the SI plane was oriented in the superior direction (76%), in the AP plane, it was in the posterior direction (63%), and in the RL plane, it was in the right direction (61%). No trends or relationships were noted between patients and direction or size of motion; however, patients 3, 4 and 11 were prone to higher levels of respiratory motion.

Based on the intrafraction motion analysis, the adjusted PTV margins were 2 mm in RL and SI planes and 3 mm in the AP plane. The average adjusted PTV structure (411.2 cm^3^), with a 2–3 mm margin, showed a reduction of 43.1 cm^3^ from the average original clinical PTV (454.3 cm^3^) with a 3–5 mm margin. Evaluation of the adjusted PTV indicated that, based on observed motion, adjusted PTV volumes would remain within the specified range for 84.6% of the imaging time. In contrast, the assessment of the original clinical PTV showed that it remained within the specified margin for 92.8% of the imaging time.

In the assessment of 20 newly generated plans utilising adjusted PTV structures (Table 2), superior dosimetric outcomes were consistently achieved. Notably, the adjusted margin plan yielded average improvements of 1.5 Gy in CTV D99%, 4.9 Gy in PTV D99% and 1.2 Gy in PTV‐high D90%. Reductions in maximum doses for OARs ranged from 1.8 Gy to 3.9 Gy. There were marked decreases in volumes receiving clinically significant doses, with V30 Gy volumes reduced to as low as 0.2 cm^3^ (from 0.6 cm^3^ for the stomach). The largest enhancements in V20 Gy were observed in the jejunum and ileum.

**Table 2:** Mean dose and range achieved by the 20 new and original margin plans.

Structure	Objective	Original Plan	New Plan
		Mean (range)	Mean (range)
Clinical target volume	D99% >33 Gy	34.3 Gy (26.8–45.5 Gy)	35.8 Gy (27.1–48.9 Gy)
Planning target volume (PTV)	D99% >30 Gy	28.7 Gy (22.7–34.6 Gy)	33.6 Gy (24.9–40.3 Gy)
	Dmax <65 Gy	66.2 Gy (62.2–72.5 Gy)	64.2 Gy (58.0–72.0 Gy)
PTV‐high	D90% >50 Gy	45.7 Gy (37.8–50.4 Gy)	46.6 Gy (39.4–52.3 Gy)
Duodenum	Dmax <33 Gy	31.6 Gy (22.7–34.8 Gy)	30.8 Gy (18.6–34.4 Gy)
	V30 Gy <5 cm^3^	0.9 cm^3^ (0–5.5 cm^3^)	0.5 cm^3^ (0–2.4 cm^3^)
	V20 Gy <20 cm^3^	19.0 cm^3^ (0–39.2 cm^3^)	15.4 cm^3^ (0–35.1 cm^3^)
Stomach	Dmax <33 Gy	30.6 Gy (17.2–33.5 Gy)	27.7 Gy (12.3–32.2 Gy)
	V30 Gy <5 cm^3^	0.6 cm^3^ (0–1.9 cm^3^)	0.2 cm^3^ (0–1.5 cm^3^)
	V20 Gy <20 cm^3^	10.1 cm^3^ (0–36.0 cm^3^)	6.3 cm^3^ (0–25.6 cm^3^)
Jejunum and ileum	Dmax <33 Gy	31.7 Gy (25.2–33.7 Gy)	29.1 Gy (23.8–32.6 Gy)
	V30 Gy <5 cm^3^	0.6 cm^3^ (0–1.9 cm^3^)	0.4 cm^3^ (0–0.9 cm^3^)
	V20 Gy <20 cm^3^	25.2 cm^3^ (0–40.8 cm^3^)	16.9 cm^3^ (0–31.5 cm^3^)
Colon	Dmax <35 Gy	29.3 Gy (17.6–32.1 Gy)	25.4 Gy (15.6–31.8 Gy)

Dmax, maximum dose; Dx%, dose received by x% of the structure; Gy, Gray; PTV‐high, PTV minus critical structures; VxGy, the percentage of the volume receiving more than xGy.

## Discussion

This study assessed the intrafraction motion in pancreatic cancer to generate adjusted planning PTV margins required for online adaptive radiotherapy treatment using abdominal compression on the MR‐Linac. The observed intrafraction motion, ranging from 1 to 3 mm in this investigation, is marginally smaller than reported in comparable studies on pancreatic cancer using abdominal compression (1.4 to 4.2 mm).^19^, ^20^ The efficacy of abdominal compression in minimising tumour motion is supported not only in pancreatic cancer but also in other abdominal cancers including adrenal gland^27^ and liver.^28^

Results from this study indicated that the adjusted PTV with new 2–3 mm margins remained within the specified range 85% of the imaging time, while the clinical PTV margin of 3–5 mm remained within the specified range for approximately 93% of the time. Grimbergen et al.,^29^ reported minimal dosimetric impact of intrafraction motion in the majority of patients treated with a 2–3 mm PTV margin. Although no correlation was observed between fraction number and tumour motion in our study, cases with outlying intrafraction motion averages highlighted that certain patients were more prone to higher levels of respiratory motion. Therefore, while abdominal compression has proven effective, implementing additional strategies to limit respiratory motion using gating could be beneficial for some patients.

The clinical application of gating on MR‐Linacs has demonstrated advantages by enabling safe margin reduction when tumours are within the specified parameters.^30^ The MR‐Linac used in this study allowed users to manually pause treatment when tumour tracking detected volumes outside predefined parameters.^31^ More recently, the clinical implementation of automatic motion management has become possible on the Unity MR‐Linac system, where, in addition to real‐time gating, the motion management system allows correction for tumour drifts. Early reports using this system indicate that gating and baseline shifts improve congruence to the planned dose to tumours in the upper abdomen, addressing both intended target coverage and OAR sparing.^29^

The reduction in PTV sizes observed in this study led to an overall improvement in target coverage and reduced doses to the OARs. Previous studies have also reported similar findings in liver^32^ and rectal^33^ cancers. Reduced margins also allow the potential to increase planned doses to target volumes and facilitate further dose escalation due to overall lower OAR doses.^34^ Dose escalation using an isotoxic approach is a promising practice in radiotherapy.^12^, ^35^, ^36^, ^37^ This involves escalating the dose to tumour volumes and increasing BED, until thresholds of OAR toxicity are reached. Our study, characterised by a reduction in dose to OARs, supports the feasibility of achieving an increase in BED. This is plausible since target volume doses can be further escalated, taking advantage of the reduced margins, thereby placing OARs at a safer distance from dose levels that may increase toxicity. This strategic reduction in margins not only contributes to improved treatment outcomes but also aligns with the trend of optimising dose escalation strategies in radiotherapy.

There are several limitations to this study. The use of abdominal compression limited this study to examine the effects of only one type of respiratory motion management. This study could be strengthened by comparing different motion management techniques such as respiratory gating deep inspiration and expiration breath‐hold techniques. Additionally, this study set a minimum time for motion data collection during treatment to 2 min. Given that treatment delivery can take up to 15 min, the motion data collected for margin creation may not reflect the total treatment time. This means that any motion occurring outside the imaging time was not reported. Furthermore, this study did not take into account patient comorbidities, which may affect respiratory motion for some patients. Despite these limitations, this work represents an important step towards advancing adaptive pancreatic cancer treatment strategies. The data and analyses presented in this study hold the potential to pave the way for the initiation of future prospective studies and clinical trials to investigate margin reduction.

## Conclusion

This study has shown that intrafraction motion is small in pancreatic SABR treatment using MR‐Linac and abdominal compression. The resulting adjusted PTV margin (2–3 mm) is smaller than the current clinical margin (3–5 mm) and led to improvements in dose metrics. However, it is crucial to exercise caution when considering the adoption of reduced margins to avoid geographical misses during treatment and should only be used if adequate resources are available, such as motion monitoring and adaptive capabilities.

## Conflict of Interest

GenesisCare and Elekta AB (Stockholm, Sweden) have a strategic research agreement.

## Data Availability

The data that support the findings of this study are available on request from the corresponding author. The data are not publicly available due to privacy or ethical restrictions.
